# Prognostic Value of Enterography Findings in Crohn’s Disease: A Systematic Review and Meta-Analysis

**DOI:** 10.3390/jimaging11110392

**Published:** 2025-11-05

**Authors:** Felipe Montevechi-Luz, Adrieli Heloísa Campardo Pansani, Juliana Delgado Campos Mello, Ana Emilia Carvalho de Paula, Lívia Moreira Genaro, Marcia Carolina Mazzaro, Daniel Lahan-Martins, Raquel Franco Leal

**Affiliations:** 1 Inflammatory Bowel Disease Research Laboratory, Gastrocenter, Colorectal Surgery Unit, Surgery Department, School of Medical Sciences, University of Campinas (Unicamp), Campinas 13083-878, SP, Brazilliviamgenaro@gmail.com (L.M.G.);; 2 Healthy Sciences Institute, Federal University of Jataí (UFJ), Jataí 75804-068, GO, Brazil; 3 Department of Radiology and Oncology, School of Medical Sciences, University of Campinas (Unicamp), Campinas 13083-878, SP, Brazil

**Keywords:** Crohn’s disease, magnetic resonance enterography, computed tomography enterography, prognosis, meta-analysis

## Abstract

Crohn’s disease is a chronic inflammatory disorder with variable progression that often leads to hospitalization, treatment escalation, or surgery. While clinical and endoscopic indices guide disease monitoring, cross-sectional enterography provides unique visualization of transmural and extramural inflammation, offering valuable prognostic information. This systematic review and meta-analysis examined the prognostic significance of magnetic resonance enterography (MRE) and computed tomography enterography (CTE) in Crohn’s disease. Following PRISMA guidelines and a registered protocol, eight databases were systematically searched through August 2024. Two reviewers independently conducted data extraction, risk-of-bias assessment (QUADAS-2), and certainty grading (GRADE). Random-effects models were applied for pooled analyses. Eleven studies, including more than 1500 patients, met eligibility criteria. Across cohorts, transmural healing on enterography was consistently associated with favorable long-term outcomes, including a markedly lower need for surgery and hospitalization. Conversely, stenosis and persistent inflammatory activity identified patients at substantially higher risk of surgery, treatment intensification, or disease-related hospitalization. The certainty of evidence was high for surgical outcomes and moderate to low for other endpoints. Conventional enterography provides meaningful prognostic insight into Crohn’s disease and should be considered a complementary tool for risk stratification and treatment planning. Transmural healing represents a protective marker of a favorable disease course, whereas structural and inflammatory findings indicate patients who may benefit from closer monitoring or earlier therapeutic intervention.

## 1. Introduction

Crohn’s disease is a chronic inflammatory bowel disorder with a heterogeneous and unpredictable course, most commonly affecting the terminal ileum and colon in young individuals during their productive years. Approximately half of patients require surgery within the first decade after diagnosis, and 70–80% undergo surgery during their lifetime. Even after apparently successful interventions, many patients experience inflammatory recurrence or require additional lines of immunosuppressive therapy [1,2]. Early identification of individuals at higher risk of complications is therefore essential to support timely and personalized therapeutic strategies.

Assessment of disease activity and treatment response has traditionally relied on clinical, endoscopic, and laboratory parameters. However, these approaches have significant limitations, particularly in assessing small bowel segments beyond the reach of endoscopy and in detecting persistent transmural inflammation, which may precede clinically relevant events even in the absence of symptoms [3,4]. In this context, magnetic resonance enterography (MRE) and computed tomography enterography (CTE) have become indispensable tools, enabling the evaluation of mural and extramural disease involvement, the identification of structural complications, and the monitoring of therapeutic response with minimal radiation exposure [5,6,7].

Recent studies have highlighted the prognostic value of enterography. Imaging features such as strictures, ulcerations, prestenotic dilatation, and active inflammation have been associated with increased risks of hospitalization, therapeutic escalation, and surgery [8,9]. Conversely, transmural healing, defined as normalization of structural abnormalities on cross-sectional imaging, has emerged as a potential therapeutic target and surrogate marker of a favorable disease course [10,11]. Despite this growing interest, however, the available evidence remains fragmented, with substantial heterogeneity in study designs, radiologic definitions, and clinical outcomes.

In light of these gaps, we performed a systematic review and meta-analysis to synthesize and critically appraise the available evidence regarding the prognostic role of enterography (MRE or CTE) in Crohn’s disease. The primary aim was to provide a robust quantitative estimate of the association between conventional radiologic findings—specifically inflammation, stenosis, and transmural healing—and major clinical outcomes, including hospitalization, therapeutic escalation, and surgery. A secondary aim was to identify methodological limitations and knowledge gaps that should guide future research.

## 2. Materials and Methods

### 2.1. Guidelines and Protocol Registration

This systematic review and meta-analysis were conducted in accordance with the PRISMA 2020 (Preferred Reporting Items for Systematic Reviews and Meta-Analyses) statement. The protocol was prospectively registered in PROSPERO (International Prospective Register of Systematic Reviews; CRD420250644775). The study selection process was documented using a PRISMA 2020 flow diagram. The completed PRISMA 2020 checklist is available in the Supplementary Material.

Because the study was based exclusively on previously published data without direct patient involvement, additional approval by an institutional ethics committee was not required.

### 2.2. Data Sources and Search Strategy

A comprehensive search was conducted in PubMed, PubMed Central (PMC), Embase (Elsevier, Amsterdam, The Netherlands), Scopus, Web of Science, ProQuest, and DeCS, covering publications from inception through 27 August 2024. The strategy combined controlled descriptors (MeSH and Emtree) with free-text terms related to Crohn’s disease, magnetic resonance enterography (MRE), computed tomography enterography (CTE), and prognostic outcomes, using Boolean operators (AND, OR). Complete search strategies for each database are provided in Supplementary File S1. In addition to electronic searches, the reference lists of eligible studies were manually screened. Deduplication was performed using Rayyan.

### 2.3. Eligibility Criteria

Studies published between 2009 and 2024, in English and available in full text, were included if they evaluated MRE or CTE for predicting clinical or surgical outcomes in Crohn’s disease. Eligible designs comprised observational studies (prospective or retrospective cohorts, case–control studies) and clinical trials that reported extractable data on associations between radiologic findings and clinical outcomes.

Exclusion criteria included studies with inadequate design, poorly defined populations, lack of extractable quantitative data, duplicate publications, systematic reviews, case reports, editorials, letters to the editor, and other non-original articles.

The review question was structured using the PICO framework: in patients with Crohn’s disease (P), are structural findings identified by MRE or CTE (I) associated with adverse clinical outcomes (O), compared with the absence or lower degree of such findings (C). This framework guided the development of eligibility criteria, data extraction, and synthesis. Only original studies reporting extractable quantitative data were eligible for meta-analysis. Reporting by sex/gender was not applicable to this aggregate review.

We specifically focused on imaging predictors identified by cross-sectional enterography (MRE/CTE), rather than clinical or genetic prognostic factors. This approach was chosen to synthesize evidence on the predictive value of enterographic features, such as transmural healing, stenosis, and inflammatory activity, in predicting primary clinical outcomes, including hospitalization, therapeutic escalation, and surgery.

### 2.4. Study Selection

Two reviewers independently screened records and extracted data. Disagreements were resolved by consensus or consultation with a third reviewer. Full texts of potentially eligible articles were assessed, yielding 43 studies for qualitative synthesis, of which 11 provided sufficient quantitative data for inclusion in the meta-analysis. Table 1 summarizes all studies included in the meta-analysis.

### 2.5. Data Extraction

Data were independently extracted by two reviewers using a standardized spreadsheet. The following variables were collected: author, year of publication, country, study design, sample size, population characteristics, imaging modality (MRE, CTE, or CTE with water enema [CTE-WE]), radiologic findings assessed, clinical outcomes analyzed (recurrence, surgery, hospitalization, therapeutic escalation, or treatment failure), follow-up duration, concomitant therapies (with emphasis on biologic agents), and reported measures of association (odds ratios, hazard ratios, relative risks, sensitivity, specificity, and 95% confidence intervals). Discrepancies were resolved by consensus. Complete data extraction tables are provided in the Supplementary Material.

### 2.6. Methodological Quality and Certainty of Evidence Assessment

Risk of bias was assessed using the QUADAS-2 tool, which evaluates both risk-of-bias and applicability domains. Two reviewers independently performed the assessments, resolving disagreements by consensus. The certainty of evidence was rated using the GRADE approach for each synthesis (meta-analyses and narrative summaries), considering the risk of bias, inconsistency, imprecision, indirectness, and publication bias. Classifications were summarized in subgroup-specific tables. Individual QUADAS-2 and GRADE assessments are provided in the Supplementary Material.

### 2.7. Data Synthesis and Statistical Analysis

Quantitative data considered comparable across studies were included in subgroup-specific meta-analyses using a random-effects model. Analyses were conducted with R software (version 4.3.2). Measures of association (relative risk, odds ratio, or hazard ratio) were calculated with 95% confidence intervals. Statistical heterogeneity was assessed using the I^2^ statistic and between-study variance (τ^2^). The robustness of pooled estimates was evaluated using leave-one-out sensitivity analyses, and potential publication bias was assessed by visual inspection of funnel plots. Formal tests for small-study effects (Egger’s test) were not performed when fewer than ten studies were available.

Of the 43 studies included in the qualitative review, 12 were eligible for quantitative synthesis and were incorporated into the meta-analyses. Studies that could not be statistically pooled were summarized narratively, organized by clinical subgroup or outcome, with emphasis on key findings and methodological limitations.

Because our objective was to evaluate prognostic rather than diagnostic performance, MRE and CTE data were analyzed together under the rationale that both modalities assess the same structural and transmural features (inflammation, stenosis, and healing) relevant to long-term outcomes. Nonetheless, to evaluate potential modality-related bias, we conducted a sensitivity analysis excluding CTE-only cohorts. The direction and magnitude of pooled effects remained stable, confirming that the inclusion of both modalities did not alter the overall estimates or conclusions.

### 2.8. Assessment of Small-Study Effects

In keeping with standard guidance, we did not perform Egger’s regression or related tests when fewer than ten studies contributed to a synthesis, as these tests are underpowered and can be unreliable under heterogeneity. Instead, we inspected funnel plots qualitatively, reported between-study variance (τ^2^/I^2^), and conducted leave-one-out analyses. When funnel-plot asymmetry was suggested, we interpreted estimates cautiously and evaluated robustness through influence analyses.

## 3. Results

### 3.1. Study Selection and Characteristics of Included Studies

**Figure 1:**
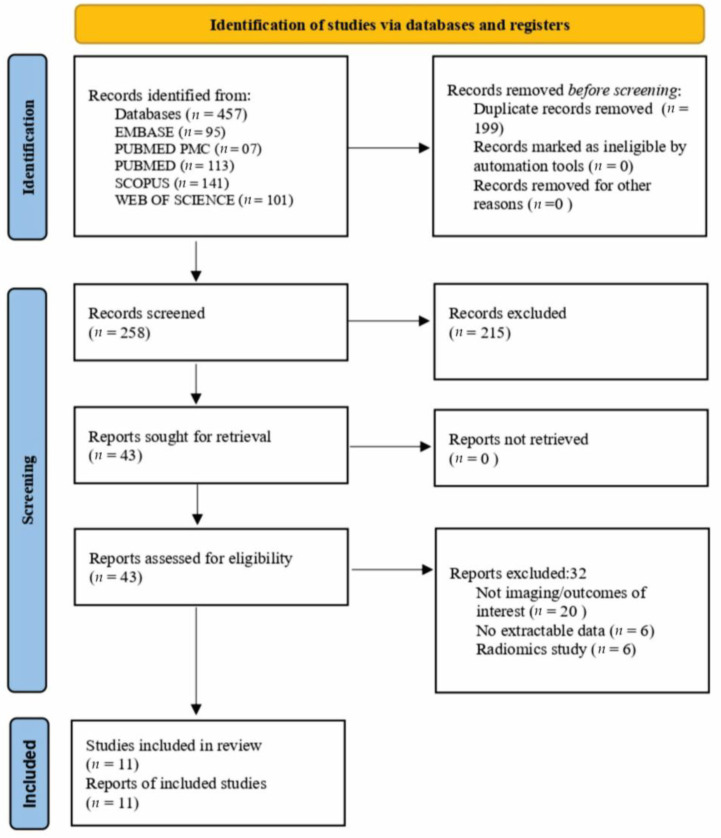
PRISMA 2020 flow diagram of study selection. The search identified 1113 records, of which 199 duplicates were removed. After screening 914 unique records, 43 full-text articles were assessed, and 11 studies were included in the systematic review and meta-analysis.

The search across eight databases identified 1113 records. After removing 199 duplicates, 914 unique records were screened. Based on titles and abstracts, 258 articles were selected for detailed evaluation, of which 43 underwent full-text review. Ultimately, 11 studies comprising 1507 patients fulfilled the eligibility criteria and were included in the systematic review and meta-analysis. The other 32 full-text articles were excluded after a detailed review, primarily because they did not evaluate the predefined imaging–outcome pairs (*n* = 20), lacked extractable quantitative data (*n* = 6), or focused on radiomic models that were not comparable with conventional imaging findings (*n* = 6). The study selection process is illustrated in the PRISMA 2020 flow diagram (Figure 1). Detailed study characteristics, including population features, imaging modalities, radiologic definitions, outcomes, and therapeutic exposures, are summarized in Supplementary File S2.

### 3.2. Transmural Healing and Surgery

**Figure 2:**
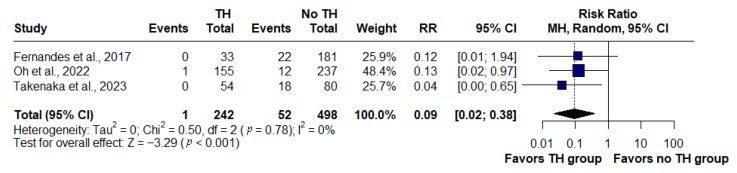
Forest plot of the association between transmural healing (TH) on magnetic resonance enterography and risk of surgery in Crohn’s disease, based on studies by Fernandes et al., 2017 [14]; Oh et al., 2022 [19]; and Takenaka et al., 2023 [20]. Patients with TH had a significantly lower likelihood of requiring surgery compared with those without TH (RR: 0.09, 95% CI: 0.02–0.38, *p* < 0.001; I^2^ = 0%).

In the pooled analysis of three studies (*n* = 740), patients who achieved transmural healing (TH) on MRE had a substantially lower risk of requiring surgery compared with those without TH. Only one surgical event occurred in the TH group (*n* = 242) versus 52 in the no-TH group (*n* = 498). The pooled risk ratio was 0.09 (95% CI: 0.02–0.38, *p* < 0.001), corresponding to a 91% reduction in relative risk. No heterogeneity was observed across studies (I^2^ = 0%) (Figure 2).

### 3.3. Transmural Healing and Hospitalization

**Figure 3:**
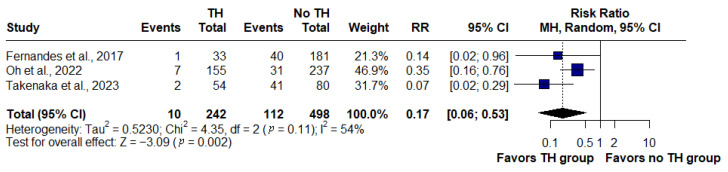
Forest plot of the association between transmural healing (TH) on magnetic resonance enterography and risk of hospitalization in Crohn’s disease, based on studies by Fernandes et al., 2017 [14]; Oh et al., 2022 [19]; and Takenaka et al., 2023 [20]. Patients with TH had a significantly lower risk of hospitalization compared with those without TH (RR: 0.17, 95% CI: 0.06–0.53, *p* = 0.002; I^2^ = 54%).

In the pooled analysis of three studies (*n* = 740), patients who achieved transmural healing (TH) on MRE had a significantly lower risk of hospitalization compared with those without TH. Hospitalization occurred in 10 of 242 patients with TH versus 112 of 498 patients without TH. The summary risk ratio was 0.17 (95% CI: 0.06–0.53, *p* = 0.002), corresponding to an 83% relative risk reduction. Moderate heterogeneity was observed across studies (I^2^ = 54%) (Figure 3).

### 3.4. Stenosis and Surgery

**Figure 4:**
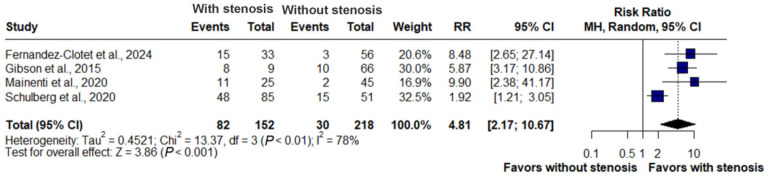
Forest plot of the association between stenosis on enterography and risk of surgery in Crohn’s disease, based on studies by Fernandez-Clotet et al., 2024 [22]; Gibson et al., 2015 [13]; Mainenti et al., 2020 [16] and Schulberg et al., 2020 [17]. Patients with stenosis had a significantly higher risk of requiring surgery compared with those without stenosis (RR: 4.81, 95% CI: 2.17–10.67, *p* < 0.001, I^2^ = 78%).

In the pooled analysis of four studies (*n* = 370), the presence of stenosis on enterography was strongly associated with an increased risk of surgery. Among 218 patients with stenosis, 82 underwent surgery, compared with 30 of 152 patients without stenosis. The summary risk ratio was 4.81 (95% CI: 2.17–10.67, *p* < 0.001), indicating an approximately fivefold higher risk. Substantial heterogeneity was observed across studies (I^2^ = 78%) (Figure 4).

### 3.5. Inflammation and Hospitalization

**Figure 5:**
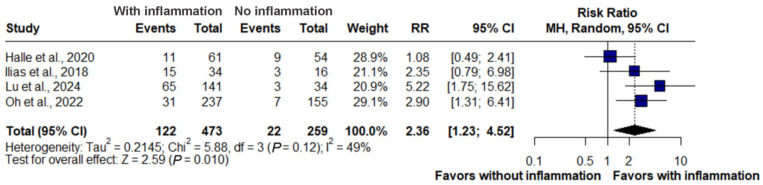
Forest plot of the association between inflammatory activity on magnetic resonance enterography and risk of hospitalization in Crohn’s disease, based on studies by Halle et al., 2024 [18]; llias et al., 2015 [15]; Lu et al., 2020 [21] and Oh et al., 2020 [19]. Patients with signs of inflammation had a significantly higher risk of hospitalization compared with those without inflammation (RR: 2.36, 95% CI: 1.23–4.52, *p* = 0.010, I^2^ = 49%).

The pooled analysis of four studies (*n* = 732) showed that the presence of inflammatory signs on MRE was significantly associated with an increased risk of hospitalization. Hospitalization occurred in 122 of 259 patients with inflammation compared with 22 of 473 patients without inflammation. The summary risk ratio was 2.36 (95% CI: 1.23–4.52, *p* = 0.010). Effect estimates across individual studies ranged from 1.08 to 5.22. Moderate heterogeneity was observed (I^2^ = 49%), reflecting variability in study design and definitions, but the overall association remained consistent (Figure 5).

### 3.6. Inflammation and Therapeutic Escalation

**Figure 6:**
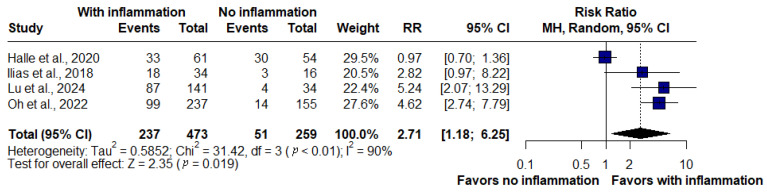
Forest plot of the association between inflammatory activity on magnetic resonance enterography and risk of therapeutic escalation in Crohn’s disease, based on studies by Halle et al., 2024 [18]; llias et al., 2015 [15]; Lu et al., 2020 [21] and Oh et al., 2020 [19]. Patients with active inflammation were significantly more likely to require treatment escalation compared with those without inflammation (RR: 2.71, 95% CI: 1.18–6.25, *p* = 0.019, I^2^ = 90%).

A pooled analysis of four studies (*n* = 732) revealed that active inflammation on MRE was significantly associated with an increased risk of therapeutic escalation. Escalation occurred in 237 of 473 patients with inflammation compared with 51 of 259 patients without inflammation. The summary risk ratio was 2.71 (95% CI: 1.18–6.25, *p* = 0.019), indicating that patients with inflammatory findings were nearly three times more likely to require treatment intensification. Substantial heterogeneity was observed across studies (I^2^ = 90%, τ^2^ = 0.5852, *p* < 0.01) (Figure 6).

### 3.7. Inflammation and Surgery

**Figure 7:**
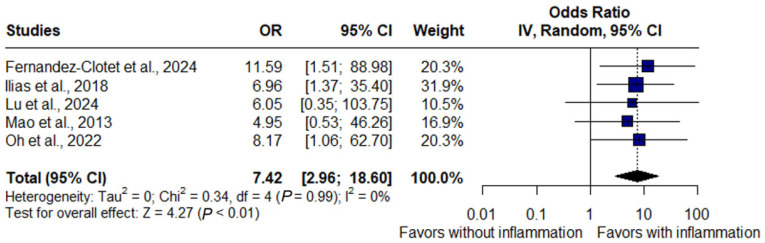
Forest plot of the association between inflammatory activity on magnetic resonance enterography and risk of surgery in Crohn’s disease, based on studies by Fernandez-Clotet et al., 2024 [22]; llias et al., 2015 [15]; Lu et al., 2020 [21]; Mao et al., 2013 [12] and Oh et al., 2020 [19]. Patients with inflammation had a significantly higher likelihood of undergoing surgery compared with those without inflammation (OR: 7.42, 95% CI: 2.96–18.60, *p* < 0.01, I^2^ = 0%).

The pooled analysis of five studies demonstrated that the presence of inflammatory signs on MRE was strongly associated with the need for surgery in Crohn’s disease. Patients with inflammation had more than a sevenfold higher likelihood of undergoing surgery compared with those without inflammation (OR: 7.42, 95% CI: 2.96–18.60, *p* < 0.01). No heterogeneity was detected across studies (I^2^ = 0%, *p* = 0.99), indicating a consistent effect (Figure 7).

### 3.8. Exploring Heterogeneity: Stenosis and Surgery

**Figure 8:**
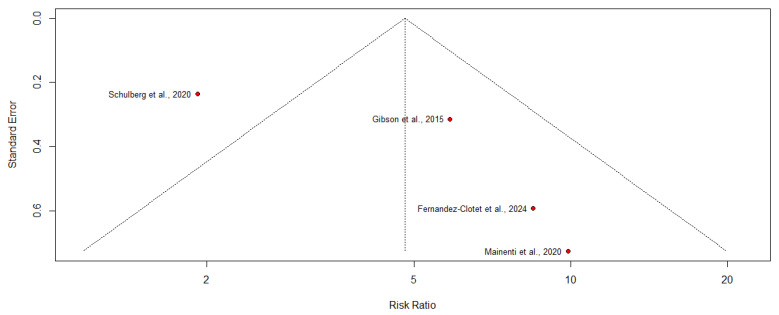
Funnel plot of studies evaluating the association between stenosis on enterography and risk of surgery in Crohn’s disease, based on studies by Schulberg et al., 2024 [17]; Gibson et al., 2015 [13]; Fernandez-Clotet et al., 2024 [22] and Mainenti et al., 2020 [16]. Some asymmetry was observed, with smaller studies tending to report larger effect sizes.

The funnel plot suggested some asymmetry, with smaller studies tending to report larger effect sizes. However, since only four studies were included in the meta-analysis, formal statistical tests for small-study effects (e.g., Egger’s test) were not performed, given their limited power with fewer than 10 studies (Figure 8).

**Figure 9:**
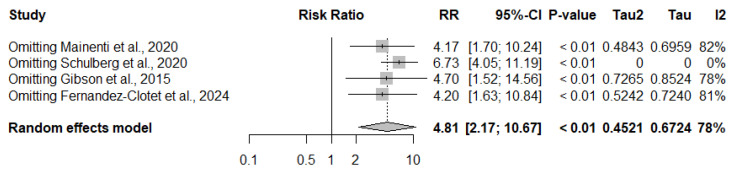
Leave-one-out sensitivity analysis for the association between stenosis and risk of surgery, based on studies by Mainenti et al., 2020 [16]; Schulberg et al., 2024 [17]; Gibson et al., 2015 [13] and Fernandez-Clotet et al., 2024 [22]. The pooled effect remained significant regardless of which study was excluded. The omission of Schulberg et al. (2020) [17] produced the largest effect estimate and eliminated heterogeneity (I^2^ = 0%).

Leave-one-out sensitivity analysis confirmed the robustness of the findings. Excluding individual studies yielded pooled risk ratios from 4.17 to 6.73, with all 95% CIs excluding 1. Omitting the most influential study [17] resulted in the largest effect estimate (RR: 6.73, 95% CI: 4.05–11.19, *p* < 0.01) and eliminated heterogeneity (I^2^ = 0%), indicating a substantial contribution to between-study variability (Figure 9).

Given k = 4 studies, formal small-study tests were not undertaken. Although the funnel plot suggested asymmetry, the association remained significant. It became more homogeneous in leave-one-out analyses, indicating that any small-study bias is unlikely to explain the pooled effect fully.

### 3.9. Exploring Heterogeneity: Inflammation and Therapeutic Escalation

**Figure 10:**
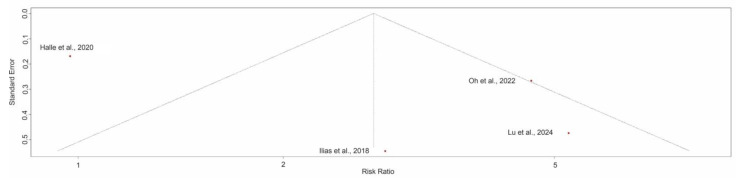
Funnel plot of studies evaluating the association between inflammatory activity on magnetic resonance enterography and therapeutic escalation in Crohn’s disease, based on studies by Halle et al., 2020 [18]; llias et al., 2018 [15]; Oh et al., 2015 [19] and Lu et al., 2024 [21]. Some asymmetry was observed, possibly reflecting publication bias or heterogeneity among studies.

The funnel plot suggested some asymmetry, which may potentially reflect publication bias or heterogeneity among studies. However, as fewer than 10 studies were available, Egger’s test was not performed due to limited validity under these conditions (Figure 10).

**Figure 11:**
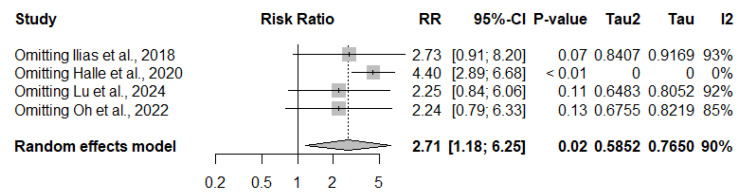
Leave-one-out sensitivity analysis for the association between inflammation on magnetic resonance enterography and therapeutic escalation, based on studies by llias et al., 2018 [15]; Halle et al., 2020 [18]; Lu et al., 2024 [21] and Oh et al., 2015 [19]. The exclusion of Hallé et al. (2020) [18] yielded the highest pooled effect estimate and eliminated heterogeneity (I^2^ = 0%), confirming the robustness of the association.

Leave-one-out sensitivity analysis demonstrated that the association remained significant regardless of which study was excluded. Notably, omitting the most influential study [18] yielded the highest pooled effect estimate (RR: 4.40, 95% CI: 2.89–6.68, *p* < 0.01) and eliminated heterogeneity (I^2^ = 0%). This finding indicates that a single study contributed substantially to the high heterogeneity observed in the overall analysis (I^2^ = 90%). Importantly, even after its exclusion, the association between inflammatory findings on MRE and therapeutic escalation remained statistically significant, reinforcing the robustness of the overall effect (Figure 11).

Because k = 4 studies contributed to this synthesis, we did not apply Egger’s regression. The effect persisted—and heterogeneity abated—after removing the most influential study, suggesting that while small-study bias cannot be excluded, it is unlikely to negate the observed association.

### 3.10. Exploring Sources of Heterogeneity

To further examine the substantial variability observed for therapeutic escalation and hospitalization, we performed exploratory subgroup analyses and qualitative meta-regression using available study-level covariates. The moderators tested included radiologic definition of inflammation (transmural healing vs. deep healing criteria), study design (prospective vs. retrospective), and predominant treatment setting (biologic-exposed vs. biologic-naïve cohorts). Differences in the definition of inflammation accounted for most of the observed heterogeneity, as studies defining inflammation by residual transmural activity (rather than combined endoscopic–radiologic criteria) showed greater effect sizes but also wider dispersion. Similarly, prospective designs with shorter follow-up periods tended to report stronger associations between inflammation and therapeutic escalation. Despite these variations, the direction of the association remained consistent across all subgroups, confirming that persistent inflammation on cross-sectional imaging is a robust predictor of unfavorable outcomes.

Additional exploratory subgroup analyses were also conducted by imaging modality (MRE vs. CTE), predominant disease location (ileal vs. ileocolonic), and study design (prospective vs. retrospective). These factors did not materially change the pooled estimates, indicating that the prognostic direction and relative magnitude of the associations were stable across methodological and clinical strata.

### 3.11. Methodological Quality Assessment (QUADAS-2)

The QUADAS-2 risk-of-bias assessment indicated a low risk across most domains in the 11 included studies. Only one study raised concerns regarding the reference standard domain, but this did not materially influence the overall appraisal. Detailed results are provided in the Supplementary File S3.

### 3.12. Certainty of the Evidence (GRADE)

#### 3.12.1. Inflammation Versus No Inflammation

Across observational cohorts using MRE/CTE, active inflammation was consistently associated with worse outcomes, but certainty varied by endpoint. For hospitalization, certainty was rated as low (RR: 2.36, 95% CI: 1.23–4.52), due to inconsistency arising from heterogeneous definitions of “inflammation” and follow-up, despite a coherent direction of effect; the absolute effect corresponded to roughly 116 more events per 1000 patients (ranging from 20 to 299 more). For therapeutic escalation, certainty was rated as very low (RR: 2.71, 95% CI: 1.18–6.25) mainly due to very high heterogeneity across designs and outcome definitions, even after considering upgrading for the magnitude of effect; the absolute difference was about 337 more per 1000 (from 35 to 1000 more). By contrast, the evidence for surgery achieved high certainty, supported by a large and homogeneous association (OR: 7.42, 95% CI: 2.96–18.60, I^2^ = 0%). All three outcomes were rated as critical to decision-making; domain-level judgments appear in Supplementary File S4.

#### 3.12.2. Stenosis Versus No Stenosis

The pooled evidence from four observational studies showed that the presence of stenosis on MRE/CTE was associated with a markedly increased risk of surgery in Crohn’s disease (RR: 4.81, 95% CI: 2.17–10.67). The certainty of evidence was rated low, downgraded one level due to inconsistency (I^2^ = 78%), reflecting variability in the magnitude of effect, although the direction of association was consistent across studies. Sensitivity analysis revealed that exclusion of the study by Schulberg et al. [17] reduced heterogeneity and produced an even stronger effect size, further supporting the robustness of the finding. Given the large magnitude of effect, the certainty was upgraded, but overall remained low. The absolute effect corresponds to approximately 524 additional surgical events per 1000 patients with stenosis (ranging from 161 to 1000 more). This outcome was considered critical for decision-making; domain-level judgments are detailed in Supplementary File S5.

#### 3.12.3. Transmural Healing Versus No Transmural Healing

Evidence from three observational cohorts demonstrated that transmural healing, as detected by MRE/CTE, was strongly associated with favorable outcomes. For surgery, the certainty of evidence was rated high (RR: 0.09, 95% CI: 0.02–0.38), supported by consistent findings across studies and a very large magnitude of effect, corresponding to approximately 95 fewer surgical events per 1000 patients with transmural healing (range: 102 fewer to 65 fewer). For hospitalization, the certainty was rated moderate (RR: 0.17, 95% CI: 0.06–0.53), downgraded for inconsistency (I^2^ = 54%) due to variation in definitions of transmural healing and hospitalization, as well as differences in patient populations and follow-up. Despite this, the association remained strong and clinically relevant, with approximately 187 fewer hospitalizations per 1000 patients (range 211 fewer to 106 fewer). Both outcomes were rated as critical for decision-making; domain-level judgments are provided in Supplementary File S6.

## 4. Discussion

This systematic review included 11 studies comprising 1507 patients from multiple centers, with MRE being more frequently employed than CTE. The studies assessed clinically relevant outcomes, including surgery, hospitalization, and therapeutic escalation across diverse treatment and follow-up settings. Three consistent patterns emerged from the quantitative synthesis. First, transmural healing was strongly associated with a reduced risk of adverse outcomes, particularly the need for surgery and, to a lesser extent, hospitalization. Second, the presence of stenosis on imaging demonstrated a robust and consistent relationship with surgical intervention. Third, signs of active inflammation correlated with unfavorable outcomes—including hospitalization, therapeutic escalation, and surgery—with the most consistent effect observed for surgery.

Regarding methodological quality and certainty of evidence, the QUADAS-2 indicated a low risk of bias across most domains. In contrast, GRADE rated the certainty of evidence as high for predicting surgery based on transmural healing and stenosis, moderate for hospitalization, and low for therapeutic escalation, the latter reflecting substantial heterogeneity.

In the present study, we deliberately focused on cross-sectional imaging findings obtained by MRE and CTE to explore their prognostic relevance in Crohn’s disease. This methodological choice reflects our objective to clarify the predictive value of radiologic features, such as transmural healing, stenosis, and active inflammation, in relation to key clinical outcomes. Although molecular and serologic biomarkers, as well as genetic profiles, are also important determinants of disease course, need for immunosuppressive therapy in young adults, and treatment response, they were beyond the predefined scope of our review. By isolating the imaging domain, our analysis aimed to provide a clearer understanding of the contribution of enterographic parameters to long-term disease prognosis. This focused approach may help establish imaging biomarkers as integral components of future multimodal predictive models that combine clinical, molecular, and radiologic data to guide personalized management in Crohn’s disease [23]

Historically, numerous observational studies have established that certain clinical and phenotypic variables are associated with a higher risk of surgery in Crohn’s disease. Population-based cohorts consistently identified younger age at diagnosis, ileal location, and stricturing or penetrating behavior as major predictors of unfavorable course. In contrast, smoking and the early need for corticosteroids further increased relapse and resection rates [24]. More recently, integrative models have been developed to refine prognostic stratification. The BACARDI model combined biochemical, radiologic, and genetic variables—C-reactive protein >11 mg/L, prestenotic dilatation on enterography, penetrating phenotype, prior anti-TNF exposure, and NOD2 rs2066844 mutation—achieving a clear separation of surgical-free survival curves across risk categories [25]. Likewise, the IMPACT study incorporated both clinical features and single-nucleotide polymorphisms into a machine-learning framework, in which the addition of four genetic loci (FSTL5, GIGYF2, rs13056955, and rs7660164) significantly improved the prediction of early intestinal resection within three years of diagnosis [26]. Collectively, these models highlight a progressive shift from purely clinical predictors toward multidimensional approaches that integrate genetic, biochemical, and imaging data. Despite these advances, radiologic parameters remain underrepresented in prognostic models, underscoring the relevance of studies focusing specifically on enterographic findings as independent predictors of long-term outcomes.

### 4.1. Prognostic Implications of Transmural Healing

Among the radiologic findings evaluated, transmural assessment emerged as the marker most consistently associated with long-term disease course. Although the prognostic significance of transmural healing has been previously recognized, the present meta-analysis provides quantitative confirmation of its impact across multiple studies and clinical contexts, reinforcing its consistency as a robust outcome predictor. The meta-analysis confirmed that patients achieving transmural healing had a substantially reduced risk of surgery and hospitalization. In contrast, persistent inflammation was linked to an increased likelihood of therapeutic escalation and adverse outcomes.

Patients with transmural healing showed lower rates of surgery, hospitalization, and treatment intensification compared with those achieving only mucosal healing [14]. Importantly, deep healing—defined as combined endoscopic and radiologic remission during anti-TNF therapy—was associated with significantly fewer adverse events than endoscopic healing alone [19]. Furthermore, a prospective study demonstrated that transmural remission on MRE provided greater predictive value for hospitalization and surgery than either clinical or endoscopic remission [20].

These findings are consistent across both prospective and retrospective studies conducted in diverse clinical settings. In MRE-based cohorts, transmural inflammation repeatedly emerged as an independent predictor of relapse, hospitalization, and treatment escalation—even among asymptomatic patients or those in clinical remission [27,28]. Fast-track MRE protocols further demonstrated that severe transmural inflammation was associated with early surgical intervention in up to half of cases, underscoring the technique’s practical utility for immediate risk stratification [15]. Comparable results have been reported with CTE, where transmural inflammation was associated with increased risk of hospitalization, including in patients who had achieved endoscopic remission [29].

Both MRE and CTE are validated imaging modalities for evaluating structural complications and transmural inflammation in Crohn’s disease. While MRE offers superior soft-tissue contrast, functional assessment, and the benefit of avoiding ionizing radiation, CTE provides greater spatial resolution and accessibility, facilitating detailed visualization of strictures, fistulas, and mesenteric involvement. Although this review did not aim to compare these techniques directly, most included studies employed MRE, reflecting its increasing use for longitudinal assessment and transmural disease monitoring.

Although MRE and CTE differ in acquisition parameters, contrast mechanisms, and radiation exposure, both modalities depict the same structural and transmural disease processes that determine prognosis. Given this conceptual overlap, they were analyzed jointly in our meta-analysis. Most included cohorts used MRE (approximately 80%), while CTE studies showed comparable effect directions and magnitude. A sensitivity analysis excluding CTE-only data yielded consistent pooled results, supporting the robustness of the findings and indicating that the combined analysis did not introduce significant modality-related bias.

Accumulating evidence reinforces the role of transmural healing as a robust prognostic marker in Crohn’s disease, consistently associated with fewer hospitalizations, reduced surgical requirements, and decreased risk of relapse. Across multiple cohorts, the absence of inflammatory activity on MRE—defined as transmural healing—was steadily correlated with favorable outcomes, even when compared with mucosal remission alone [21]. This distinction highlights the capacity of MRE to capture deeper resolution of intestinal inflammation. Conversely, persistence of inflammatory findings on serial imaging is associated with a twofold higher risk of surgery and nearly a threefold shorter time to event [18]. Several studies have demonstrated that transmural resolution correlates with mucosal healing and predicts a sustained favorable course. International guidelines recognize the MaRIA score as an accurate and responsive tool for monitoring therapeutic response to immunosuppressive therapy [30,31]. A recent systematic review further emphasized transmural healing as a promising therapeutic target, associated with superior outcomes and exerting greater prognostic impact than endoscopic remission alone [14].

While the protective effect of transmural healing was highly consistent for surgery (I^2^ = 0%), the analysis of hospitalization showed moderate heterogeneity (I^2^ = 54%) in our meta-analysis. This variability reflected important methodological differences. Definitions of transmural healing were not uniform: one study required concomitant normal findings on both MRE and colonoscopy [14], another employed the broader concept of deep healing (combined endoscopic and radiologic remission assessed by CTE/MRE) [19], and a third applied the sMaRIA combined with the absence of endoscopic ulcers [20]. Definitions of hospitalization also varied, ranging from the inclusion of Crohn’s-related complications and treatment-related adverse events [14] to hospitalization embedded in a composite endpoint [19] and to the restriction of the outcome to Crohn’s-related admissions under biologic therapy [20]. Populations differed substantially—from cohorts with minimal biologic exposure to those under long-term maintenance with advanced therapies—and follow-up periods ranged from 12 to 30 months. These discrepancies explained the reduced statistical consistency for hospitalization and underscored the need to standardize imaging criteria and clinical outcomes in future studies.

Beyond statistical inconsistency, the variability in defining key imaging constructs—particularly transmural healing, stenosis, and inflammation—represents a fundamental methodological limitation of the available literature. Studies applied distinct criteria for healing (MaRIA thresholds, sMaRIA normalization, or combined deep healing), for stenosis (variable lumen narrowing and prestenotic dilation cut-offs), and for inflammation (wall thickening, hyperenhancement, or combined mural/mesenteric findings). This lack of harmonization complicates quantitative synthesis and limits the comparability of prognostic performance across cohorts. Standardized imaging definitions, ideally derived from international consensus statements such as those of ECCO–ESGAR or the Society of Abdominal Radiology, are urgently needed to enable reproducible and clinically translatable prognostic assessment.

In contrast, the analysis of surgical requirements showed no heterogeneity (I^2^ = 0%). Among 740 patients, only one surgical event occurred in the transmural healing group, compared with 52 events in those without healing. The meta-analysis demonstrated a risk ratio of 0.09 (95% CI: 0.02–0.38, *p* < 0.001), corresponding to a 91% relative risk reduction in surgery. This consistency was likely explained by the fact that surgery represented a more objective endpoint, less influenced by institutional practices than hospitalization, as it reflected structural complications or therapeutic refractoriness with limited scope for interpretation across centers. Although definitions of transmural healing varied, all consistently converged in linking the absence of transmural inflammation with reduced structural progression and, consequently, lower surgical requirements. Differences in follow-up duration (12, 18, and 30 months) did not alter this protective effect, which remained stable across time horizons.

Taken together, these results indicated that while hospitalization outcomes remained influenced by methodological variability, the need for surgery emerges as a solid and uniform endpoint, reinforcing transmural healing as a consistent and clinically meaningful prognostic marker.

### 4.2. Prognostic Implications of Stenosis

The meta-analysis demonstrated a strong association between stenosis detected on enterography and the risk of surgery, with a relative risk of nearly five (RR: 4.81, 95% CI: 2.17–10.67), and no statistical heterogeneity (I^2^ = 0%).

Individual studies consistently reinforced this finding. One cohort reported that stenosis identified on MRE prior to initiation of anti-TNF therapy was associated with a markedly increased risk of surgery (HR 26.4) and a significantly shorter time to intervention, particularly when penetrating complications coexisted [13]. Another study provided quantitative evidence by demonstrating that apparent diffusion coefficient (ADC) values derived from diffusion-weighted imaging distinguished between stenoses that progressed to resection and those managed conservatively, suggesting that functional parameters may refine risk stratification [16]. Additional work identified three straightforward, simple MRE parameters—bowel wall thickness ≥ 10 mm, stricture length > 5 cm, and prestenotic dilatation ≥ 30 mm—as independent predictors of surgery, and proposed a composite risk score based on these variables, demonstrating good predictive accuracy [17]. Moreover, persistent stenosis combined with high MaRIA scores and structural complications significantly increased the likelihood of surgery, emphasizing that stenosis rarely occurs in isolation and often coexists with other markers of transmural damage [22]. However, the present meta-analysis could not distinguish between inflammatory and fibrotic strictures or assess their evolution after therapeutic adjustment, as performed by Hallé et al.’s study [18]. This distinction is clinically relevant because inflammatory strictures may respond to medical therapy, whereas fibrotic strictures are more likely to require surgical intervention.

The converging body of evidence highlighted stenosis as one of the most robust predictors of surgical risk in Crohn’s disease, yet nuances emerged when contrasting data sources. Population-based cohorts consistently demonstrate that stricturing behaviour independently drives surgery, with long-term follow-up confirming that nearly half of patients eventually required resection, regardless of therapeutic advances [32]. Surgical reviews corroborated this inevitability, emphasizing that long and fibrotic strictures were poorly responsive to medical therapy and frequently progressed despite biologic use, leading some guidelines to advocate early resection for isolated ileal disease [33].

Radiological studies refined this picture by showing that not all strictures carried the same prognostic weight. Cross-sectional imaging parameters such as stricture length, degree of prestenotic dilatation, and coexistent penetrating complications provided quantitative risk stratification. Additionally, functional imaging markers, such as ADC values, may further distinguish those who are destined for surgery [34]. Importantly, imaging-based definitions appear more predictive of adverse outcomes than endoscopic findings alone, as endoscopically defined strictures in isolation carried substantially lower risk [35].

Consensus statements on endoscopic management add another dimension, recognizing balloon dilation and stricturotomy as strategies to delay surgery in selected patients, while acknowledging that outcomes are largely dictated by the complexity of the stricture. Length beyond 4–5 cm, multiplicity, or associated dilation sharply increase the likelihood of resection [36]. This therapeutic evidence aligns with radiological prognostic markers, reinforcing that stricture morphology, rather than mere presence, determines surgical trajectory.

Taken together, these data establish a consistent narrative: stenosis represents a pivotal inflection point in Crohn’s disease, where structural damage surpasses the capacity of medical therapy. Cross-sectional imaging offers the most reliable tools for stratifying surgical risk, while endoscopic therapy may temporarily delay surgery in selected cases but cannot alter the natural history of extensive or complicated disease.

Despite the absence of statistical heterogeneity, methodological differences among studies warranted attention. One large cohort predominantly included patients with long-standing disease, multiple prior resections, and a high prevalence of prestenotic dilatation, representing a structurally advanced population [17]. This translated into a high absolute risk of early surgery, leading to the development of a dedicated radiologic score to predict unfavorable outcomes. In contrast, cohorts evaluated at earlier disease stages—often at the time of first-line biologic therapy—reported lower absolute rates of surgery, although the relative risks remained high [13,16]. Similarly, a prospective study in patients receiving biologics demonstrated that persistent stenosis combined with active inflammation and structural complications retained prognostic significance, albeit with a more moderate effect size [22].

Leave-one-out sensitivity analysis confirmed the robustness of the finding: the exclusion of the Schulberg study [17] increased the pooled estimate to an RR of 6.73 and eliminated between-study variability. This indicates that although Schulberg contributed most to heterogeneity, it did not undermine the overall consistency of the effect. Taken together, the evidence demonstrated that across diverse clinical contexts—from early disease under biologic therapy to structurally advanced cohorts with prior resections—stenosis detected on enterography remains one of the most consistent and clinically meaningful predictors of surgical requirement in Crohn’s disease. Although the association between stenosis and surgery may reflect, in part, therapeutic decision-making based on imaging and symptoms, the consistent magnitude of this effect across studies supports the role of enterography as an objective prognostic marker for identifying patients at the highest surgical risk.

While the presence of stenosis is strongly associated with surgery, it should be acknowledged that surgical intervention in Crohn’s disease is not always indicative of an unfavorable outcome. In selected patients, particularly those with localized ileal disease or adolescents with growth delay, elective surgery may be chosen as part of an individualized management strategy aimed at improving long-term outcomes.

### 4.3. Prognostic Implications of Active Transmural Inflammation

The presence of active inflammation on enterography was consistently associated with adverse clinical outcomes in Crohn’s disease, including hospitalization, therapeutic escalation, and the need for surgery [6]. In the included studies, inflammation was defined based on conventional radiologic features of activity, such as bowel wall thickening greater than 7 mm, mural hyperenhancement, edema, comb sign, lymphadenopathy, or associated complications (abscess, fistula, or stricture). In Hallé et al.’s study [13], for example, patients were considered inflamed when these findings persisted or worsened on follow-up MRE (non-responders, *n* = 61) and non-inflamed when they resolved or decreased (responders, *n* = 54). This definition was representative of the criteria applied across studies contributing to the pooled analysis. These findings reinforce that persistent inflammatory activity on imaging, even in patients in clinical follow-up, may serve as a robust prognostic marker of unfavorable disease course. The present review was not designed to perform a head-to-head comparison between MRE and CTE regarding diagnostic accuracy or therapeutic response monitoring. Both modalities are validated for assessing small-bowel disease activity, and the choice between them should consider availability, expertise, clinical context, and safety aspects such as radiation exposure and contrast use.

In this analysis, four studies evaluating hospitalization and therapeutic escalation were included (*n* = 732; 473 without inflammation, 259 with inflammation). Active inflammation was associated with a 2.36-fold higher risk of hospitalization and nearly a threefold higher risk of therapeutic escalation, underscoring the clinical impact of persistent activity across multiple management domains. For surgery, five studies consistently demonstrated that patients with inflammation had more than a sevenfold increased risk, confirming the magnitude and robustness of this marker.

The heterogeneity observed across studies evaluating hospitalization and therapeutic escalation primarily reflected methodological and population-level differences rather than true inconsistency in the underlying association. Exploratory subgroup and qualitative meta-regression analyses suggested that heterogeneity was largely explained by variability in radiologic definitions of inflammation, study design, and therapeutic context. Studies applying purely transmural criteria and shorter follow-up intervals showed stronger but more dispersed estimates, whereas mixed deep-healing definitions produced milder and more homogeneous effects. Differences in patient populations—particularly biologic exposure and disease chronicity—also contributed modestly to variance. Nonetheless, all analyses demonstrated the same directional relationship, reinforcing that persistent inflammation on enterography consistently identifies patients at higher risk for treatment intensification and hospitalization, irrespective of methodological differences.

For hospitalization, the pooled analysis demonstrated a significantly increased risk (RR: 2.36, 95% CI: 1.23–4.52; *p* = 0.010), although moderate heterogeneity was observed (I^2^ = 49%). Individual estimates ranged from an RR of 1.08 to 5.22, indicating methodological variability without loss of overall consistency. Cohort evidence aligned with this finding: one study reported that persistent transmural inflammation increased hospitalization risk compared with transmural healing [21], another in anti-TNF–treated patients reported greater hospitalization requirements when deep healing was not achieved [19], and a third in small-bowel Crohn’s disease found that lack of radiologic response did not reduce hospitalization risk despite higher surgical and endoscopic interventions [18]. In symptomatic patients, significant MRE-detected inflammation was associated with both increased hospitalization and higher surgical requirements [15].

The observed heterogeneity for hospitalization reflected differences in study design and definitions of inflammation [37]. Some cohorts compared responders with non-responders [18], while others compared transmural healing with no healing [21], or applied fast-track MRE protocols for short-term stratification [15]. In long-term biologic cohorts, deep healing criteria, which combined endoscopic and radiologic remission, were observed [19]. Variability in definitions of inflammation (ranging from significant lesions to partial response) and in patient populations (from refractory cases to biologic-naïve or maintenance cohorts) explained the moderate inconsistency, although the association remained consistent.

Therapeutic escalation was also strongly associated with inflammation. The pooled analysis revealed a nearly threefold higher risk of escalation (RR: 2.71, 95% CI: 1.18–6.25, *p* = 0.019), but heterogeneity was substantial (I^2^ = 90%). One retrospective cohort with heterogeneous treatment regimens and a broad definition of “adjustment” diluted the effect [18]. Sensitivity analysis confirmed this: the exclusion of that study reduced heterogeneity to zero and increased the pooled effect (RR: 4.40, 95% CI: 2.89–6.68), indicating that persistent inflammation predicts escalation across contexts. Funnel plot inspection suggested possible asymmetry, raising concerns about publication bias, although fewer than 10 studies precluded formal testing. Cohort evidence corroborates this pattern, with higher escalation rates in patients without deep healing [19], in those with persistent transmural inflammation [21], and in nearly all patients with significant inflammation on fast-track MRE [15]. By contrast, one cohort found no significant difference between persistent inflammation and partial radiologic responders [18].

The heterogeneity observed for escalation largely reflects conceptual differences in outcome definitions. Some studies restricted escalation to biologic intensification or switch, whereas others included immunosuppressants and corticosteroids. Population differences (biologic-naïve, long-term maintenance, or refractory) and follow-up (3–30 months) added further variability. Nevertheless, across analyses, the association remained statistically significant, reinforcing persistent inflammation as a robust marker of treatment intensification.

In contrast, the pooled analysis for surgery demonstrated high consistency with no heterogeneity (I^2^ = 0%). Patients with inflammatory activity had a nearly sevenfold higher likelihood of undergoing surgery (OR: 7.42, 95% CI: 2.96–18.60, *p* < 0.01). Cohort studies confirmed this finding: significant inflammation, particularly when associated with abscesses or fistulas, predicted higher surgical rates [15]; lack of radiologic response was linked to earlier surgery [18]; the absence of deep healing was an independent predictor of resection under anti-TNF therapy [19]; and all surgical events occurred in patients without transmural healing [21]. Postoperative studies further showed that inflammation on CTE predicted reoperation more accurately than ileocolonoscopy, especially for proximal or extramural complications [12]. Prospective cohorts also confirmed MRE-detected inflammation was a long-term predictor of surgical resection [22].

Surgery stood out not only for its strong association with inflammation but also for its reliability as an endpoint reflecting irreversible damage. Quantification of structural damage using indices such as the Lémann score proved valuable in predicting the need for surgery [38]. The absence of heterogeneity suggests that surgical indication, being less subject to institutional variability than hospitalization or escalation, provided a particularly stable endpoint. More extensive, penetrating, or extramural patterns of inflammation also increased surgical risk, anticipating disease progression. This pattern extended to the postoperative setting, where MRE or CTE outperformed ileocolonoscopy in predicting recurrence by detecting proximal and extramural complications not visible endoscopically. Clinically, persistent transmural inflammation should be interpreted as a signal of imminent therapeutic failure, warranting an early reassessment of management.

Importantly, active inflammation on MRE or CTE has been consistently associated with poor outcomes even in asymptomatic patients [27]. In a prospective study, severe MaRIA lesions, stenoses, or penetrating complications persisting after one year of biologic therapy predicted relapse, hospitalization, and surgery despite clinical remission [22]. Similarly, inflammatory features assessed two years after diagnosis were major determinants of long-term adverse outcomes [39], and lack of radiologic response doubled the risk of surgery and nearly tripled the speed to even [18].

Specific imaging patterns also demonstrated prognostic significance. In patients treated with infliximab or adalimumab, severe ulcers or strictures on MRE predicted loss of response and earlier recurrence [40]. Penetrating or mixed patterns were associated with therapeutic escalation or surgery, whereas normal MREs rarely prompted a change in treatment [41]. Internal fistulas and extramural complications correlated with poorer nutritional status and greater therapeutic requirements, underscoring their role as markers of aggressive disease phenotypes [42].

Despite strong evidence, important limitations persist. Several studies have reported a dissociation between radiologic improvement and clinical benefit, with up to one-third of patients showing reduced inflammatory activity without symptomatic relief [43,44]. The ECCO-ESPGHAN guidelines emphasize that no single modality, including MRE, reliably predicts a sustained response; instead, they recommend combined monitoring with clinical indices and biomarkers such as fecal calprotectin [45]. Comparative studies have confirmed that integrating MRE with fecal biomarkers enhances the accuracy of detecting activity and predicting complications [46,47]. However, most radiologic scores still lack robust prognostic validation and face practical limitations, including the underestimation of small-bowel involvement and limited reproducibility outside expert centers, which restricts their adoption and use as stand-alone targets [48]. Performance analyses also indicate a variable predictive value of scores, such as MaRIA, when compared with endoscopy, underscoring the need for complementary assessment [49]. Collectively, recent evidence consolidates MRE as the leading imaging modality for predicting unfavorable outcomes in Crohn’s disease, including hospitalization and surgery [50].

Beyond methodological heterogeneity, residual confounding related to treatment exposure, disease phenotype, and baseline severity represents another limitation of the available evidence. The majority of included cohorts were retrospective and often combined patients with variable disease duration and treatment histories, including differing exposure to biologics or immunosuppressants. Such imbalances may influence both imaging findings and clinical outcomes, potentially amplifying or attenuating observed associations. Although most studies performed multivariable analyses to account for key covariates, unmeasured confounders—such as disease behavior, comorbidities, and timing of imaging relative to therapeutic interventions—cannot be entirely ruled out. These considerations highlight the need for prospective, treatment-stratified cohorts with standardized imaging and uniform clinical endpoints to better delineate the independent prognostic role of enterographic features in Crohn’s disease.

### 4.4. Implications for Treat-to-Target Strategies

The findings of this review can also be interpreted within the context of evolving treat-to-target strategies in Crohn’s disease. The STRIDE-II consensus reaffirmed clinical remission and endoscopic healing as long-term targets, while identifying transmural healing as an important adjunctive marker of remission depth, particularly relevant to Crohn’s disease [51]. Our results, demonstrating that transmural healing is consistently associated with reduced risks of hospitalization and surgery, support its potential integration into this framework. Although not yet established as a formal therapeutic target, accumulating evidence suggests that cross-sectional imaging may serve as a complementary endpoint to endoscopy, aligning radiologic outcomes with the broader aim of preventing structural damage and altering the long-term course of disease. This interpretation is consistent with ECCO-ESGAR guidance, which highlights the role of MRE indices such as MaRIA for standardized assessment of disease activity and therapeutic response [31], and with recent systematic reviews underscoring transmural healing as an emerging treatment goal associated with improved long-term outcomes [11].

From a practical perspective, imaging biomarkers could be directly integrated into treat-to-target workflows as adjunctive endpoints complementing clinical and endoscopic remission. Within the STRIDE-II framework, cross-sectional imaging provides a means to assess “deep remission” by confirming transmural healing and absence of structural damage—parameters not captured by endoscopy or biomarkers alone. MRE or CTE could therefore guide therapeutic decisions at several stages: confirming treatment response before de-escalation, identifying subclinical inflammation prompting early escalation, or detecting progressive stenosis that may warrant surgical evaluation. Incorporating standardized imaging intervals (e.g., baseline, 6–12 months, and during symptomatic or biomarker relapse) could help harmonize monitoring strategies across centers. In clinical practice, this approach supports multidisciplinary decision-making, aligning radiologic, endoscopic, and biochemical data to prevent irreversible bowel damage and optimize long-term outcomes for patients with Crohn’s disease.

### 4.5. Practical Limitations for Implementation

Despite the strong prognostic value of imaging findings and their potential role within treat-to-target strategies, several practical barriers remain. High costs, heterogeneous access across healthcare systems, and the requirement for expertise concentrated in referral centers currently limit the widespread adoption of cross-sectional imaging as a therapeutic endpoint. In clinical practice, enterography should complement rather than replace clinical assessment and biochemical monitoring, being most useful when biomarker results (serum or fecal) are inconclusive or discordant with the patient’s clinical status. In addition, the cost, examination time, and potential cumulative exposure to gadolinium should be weighed against the incremental information provided by imaging. These challenges emphasize the need for solutions that integrate prognostic accuracy with real-world feasibility.

### 4.6. Future Directions

This systematic review and meta-analysis focused on the prognostic value of enterographic findings, such as transmural healing, stenosis, and active inflammation, in predicting major clinical outcomes in Crohn’s disease. While patient-specific variables, including phenotype, disease extent, and treatment history, play important roles in determining therapeutic response, these factors were beyond the scope of our analysis. Nevertheless, by highlighting imaging features associated with adverse outcomes, our findings may contribute to more individualized care strategies and support risk stratification within a treat-to-target framework.

Looking ahead, radiomics and artificial intelligence, including machine learning approaches, hold promise for overcoming current limitations in assessing Crohn’s disease. These techniques may enable the extraction of quantitative imaging biomarkers, facilitate standardized interpretation across centers, and improve the prognostic accuracy of cross-sectional imaging. By integrating advanced computational models with clinical and biological data, future research may help establish reproducible imaging endpoints that are both feasible in practice and predictive of long-term outcomes.

Radiomics studies have already shown prognostic value across different disease phases. MRE-based features from the ileal wall predicted long-term therapeutic response in pediatric patients [52]. In adults, radiomic signatures of ileal segments anticipated the need for surgery within 12 months, outperforming conventional scores [53]. CTE-based models that combine luminal and mesenteric attributes achieved robust accuracy for inflammatory activity and surgical risk, confirmed by survival and decision analyses [54,55]. Mesenteric fat signatures from preoperative CTE further predicted anastomotic recurrence after ileocolic resection, outperforming intestinal signatures as stable independent markers [56]. Nomograms integrating radiomic and clinical data also enabled prediction of mucosal healing after infliximab, with superior net clinical benefit [57].

These findings support the use of radiomics as a complementary tool for individualized risk stratification. However, the predominance of retrospective designs, methodological heterogeneity, and lack of external validation underscore the need for prospective, multicenter studies before clinical adoption.

### 4.7. Complementary Role of Intestinal Ultrasound

Intestinal ultrasound has emerged as a complementary alternative in settings where access to magnetic resonance imaging or computed tomography is limited. It is a non-invasive, widely available, and low-cost method that allows dynamic evaluation of bowel wall thickness, vascularity, and extramural complications. Although its accuracy depends on operator expertise and may vary across centers, studies have demonstrated good agreement with enterography in detecting transmural inflammation and strictures, making it a potentially valuable tool for prognostic monitoring in diverse clinical practices.

In a prospective study, ultrasound demonstrated high sensitivity and specificity for detecting ulcers and complications, with concordance nearly identical to that of magnetic resonance imaging and colonoscopy in guiding therapeutic decisions [58]. Sonographic activity has also been identified as a risk factor for relapse, whereas parietal healing was associated with a more favorable course [59]. More recently, a population-based cohort study confirmed that early transmural remission, detected by ultrasound, was associated with higher rates of sustained steroid-free remission and a reduced risk of treatment escalation [60]. Thus, intestinal ultrasound has become a feasible and cost-effective alternative for prognostic monitoring in Crohn’s disease.

### 4.8. Limitations

This study has several important limitations. The number of eligible studies and sample sizes within individual meta-analyses were relatively small, limiting statistical power for subgroup analyses. Considerable heterogeneity was present in study design, population characteristics, and radiologic definitions of transmural healing, stenosis, and inflammation. Most included cohorts were retrospective, which may introduce selection and confounding biases. In addition, although sensitivity analyses confirmed the robustness of the main findings, the possibility of publication bias cannot be excluded, as smaller negative studies may remain unpublished. These constraints highlight the need for large, prospective, and multicenter studies with standardized imaging criteria and harmonized clinical endpoints to confirm and extend the present findings.

Finally, several syntheses included fewer than ten studies, precluding adequately powered tests for small-study effects. Although funnel-plot inspection and leave-one-out analyses supported the robustness of the main findings, publication or small-study bias cannot be excluded and may have modestly inflated some estimates—particularly where funnel-plot asymmetry was observed.

### 4.9. Overall Interpretation and Clinical Perspective

Taken together, this meta-analysis advances current knowledge by providing the first quantitative synthesis to establish the magnitude and certainty of the prognostic associations between enterographic findings and key clinical outcomes in Crohn’s disease. Unlike previous narrative reviews, our results demonstrate that transmural healing confers a strong and consistent protective effect, whereas stenosis and persistent inflammation predict adverse outcomes across clinical contexts, independent of study design or population. These findings strengthen the evidence base supporting cross-sectional imaging as an objective prognostic tool, directly relevant to treat-to-target management strategies. They also provide a benchmark for future studies, guiding the selection of imaging endpoints in clinical trials and informing guideline development toward standardized, outcome-driven imaging assessment. By consolidating evidence across modalities and settings, this work bridges the gap between research and clinical application, reinforcing enterography as a cornerstone in personalized, risk-stratified management of Crohn’s disease.

## 5. Conclusions

This systematic review and meta-analysis demonstrate that conventional enterography (CTE and MRE) findings carry substantial prognostic value in Crohn’s disease. Transmural healing consistently emerged as a protective marker, strongly associated with reduced risk of surgery and hospitalization. In contrast, the presence of stenosis or persistent inflammation identified patients at markedly higher risk of surgery, hospitalization, or therapeutic escalation, with surgery providing the most robust and homogeneous endpoint across studies. These findings reinforce the role of MRE and CTE as indispensable tools for risk stratification and long-term management in Crohn’s disease. At the same time, heterogeneity across the included studies—both in radiologic criteria and in the outcomes assessed—underscores the need for future prospective research that applies standardized definitions, which may better align with international consensus statements [37,61,62]. Incorporating enterography into routine monitoring, alongside clinical and biomarker assessments, may enable more accurate prognostic stratification and support personalized therapeutic strategies aimed at preventing structural damage and improving long-term outcomes.

A key methodological limitation of current evidence lies in the inconsistent definitions of transmural healing, stenosis, and inflammatory activity across studies. The development and adoption of standardized imaging criteria would greatly improve comparability among cohorts, strengthen prognostic evidence, and support the incorporation of radiologic endpoints into treat-to-target strategies.

## Figures and Tables

**Table 1:** Summary of included references in the meta-analysis.

Reference	Year	Method Imaging Modality	Problems Solved/Advantages (Prognostic Associations)	Limitations
Mao et al. [12], China	2013	Prospective cohort; CTE (post-surgical)	Postoperative inflammatory recurrence on CTE was associated with later reoperation.	Small sample; heterogeneous postoperative therapy.
Gibson et al. [13], Ireland	2015	Retrospective cohort; 1.5 T MRE	Stricture with prestenotic dilatation strongly predicted the need for surgery.	All patients on anti-TNF; long-standing disease.
Fernandes et al. [14], Portugal	2017	Retrospective cohort; 1.5 T MRE	Transmural healing was associated with lower hospitalization and surgery risk.	Combined clinical + endoscopic definition of healing.
Ilias et al. [15], Hungary/Canada	2018	Retrospective cohort; 3 T MRE (fast-track)	Active transmural inflammation was associated with therapeutic escalation, hospitalization, and surgery.	Short follow-up (≤3 mo); heterogeneous therapy.
Mainenti et al. [16], Italy	2020	Retrospective cohort; 3 T MRE	Prestenotic dilatation > 2.5 cm predicted subsequent surgery.	One-year follow-up; mixed treatments.
Schulberg et al. [17], Australia	2020	Retrospective cohort; 1.5 T MRE	Stricture ≥ 3 cm prestenotic dilatation associated with early surgery.	Predominantly stricturing phenotype; high prior surgery rate.
Hallé et al. [18], France	2020	Retrospective cohort; 1.5 T MRE (serial)	Persistent inflammation after therapy was associated with hospitalization and escalation.	No distinction between fibrotic vs. inflammatory stricture.
Oh et al. [19], Korea	2022	Retrospective cohort; CTE/MRE after ≥ 1 yr anti-TNF	Transmural healing linked to reduced surgery/hospitalization; active inflammation predicted escalation.	Anti-TNF-only population; variable imaging modality.
Takenaka et al. [20], Japan	2023	Prospective cohort; 3 T MRE	Transmural healing (sMaRIA < 2 and SES-CD < 4) was associated with fewer hospitalizations and surgeries.	Prospective but single-center; biologic-treated cohort.
Lu et al. [21], China	2024	Retrospective cohort; 3 T MRE	Active inflammation without transmural healing was associated with hospitalization, escalation, and surgery.	Mixed biologic exposure; retrospective design.
Fernández-Clotet et al. [22], Spain	2024	Prospective cohort; 1.5 T/3 T MRE	Stricture > 3 cm upstream dilatation predicted surgery despite biologic therapy.	Two-year follow-up; small sample for multivariable analysis.

CTE: computed tomography enterography. MRE: magnetic resonance enterography. T: Tesla. TNF: tumor necrosis factor.

## Data Availability

The original contributions presented in this study are included in the article/Supplementary Materials. Further inquiries can be directed to the corresponding author.

## Supplementary Materials

The following supporting information can be downloaded at: https://www.mdpi.com/article/10.3390/jimaging11110392/s1, File S1: Search strategy, File S2: Characteristics of included studies in the meta-analysis; File S3: Risk of bias assessment (QUADAS-2); File S4: Certainty of evidence (GRADE)—Transmural healing; File S5. Certainty of evidence (GRADE)—Stenosis; File S6: Certainty of evidence (GRADE)—Inflammation; File S7: PRISMA 2020 Checklist.

## Abbreviations

The following abbreviations are used in this manuscript:

ADC	Apparent Diffusion Coefficient
AGA	American Gastroenterological Association
CD	Crohn’s Disease
CI	Confidence Interval
CTE	Computed Tomography Enterography
CTE-WE	Computed Tomography Enterography with Water Enema
ECCO	European Crohn’s and Colitis Organisation
ECCO-ESPGHAN	European Crohn’s and Colitis Organisation—European Society for Paediatric Gastroenterology Hepatology and Nutrition
ESGAR	European Society of Gastrointestinal and Abdominal Radiology
GRADE	Grading of Recommendations Assessment, Development and Evaluation
HR	Hazard Ratio
I^2^	Heterogeneity statistic (Inconsistency Index)
MaRIA	Magnetic Resonance Index of Activity
MEGS	Magnetic Resonance Enterography Global Score
MRE	Magnetic Resonance Enterography
OR	Odds Ratio
PICO	Patient/Problem, Intervention, Comparison, Outcome
PRISMA	Preferred Reporting Items for Systematic Reviews and Meta-Analyses
PROSPERO	International Prospective Register of Systematic Reviews
QUADAS-2	Quality Assessment of Diagnostic Accuracy Studies 2
RR	Risk Ratio (Relative Risk)
SAR	Society of Abdominal Radiology
STRIDE-II	Selecting Therapeutic Targets in Inflammatory Bowel Disease (Second Consensus Initiative)
TH	Transmural Healing
τ^2^	Between-study variance
