# Supplementary material for: Prognostic Value of Enterography Findings in Crohn’s Disease: A Systematic Review and Meta-Analysis
Source: J Imaging. 2025 Nov 5;11(11):392. doi: 10.3390/jimaging11110392 (PMC12653103; doi:10.3390/jimaging11110392)
Supplement: Supplementary file 1 [file jimaging-11-00392-s001.zip › Supplementary File S3.pdf]

**Supplementary File S3. Risk of bias assessment using the QUADAS-2 tool**

| **Study** | **Patient Selection** | **Index Test** | **Reference Standard** | **Flow and Timing** |
|---|---|---|---|---|
| Fernández-Clotet et al. (2024) | Low risk | Low risk | Low risk | Low risk |
| Gibson et al. (2015) | Low risk | Low risk | Low risk | Low risk |
| Mainenti et al. (2020) | Low risk | Low risk | Low risk | Low risk |
| Schulberg et al. (2020) | Low risk | Low risk | Low risk | Low risk |
| Ilias et al. (2018) | Low risk | Low risk | Unclear risk | Low risk |
| Hallé et al. (2020) | Low risk | Low risk | Low risk | Low risk |
| Lu et al. (2024) | Low risk | Low risk | Low risk | Low risk |
| Mao et al. (2013) | Low risk | Low risk | Low risk | Low risk |
| Oh et al. (2022) | Low risk | Low risk | Low risk | Low risk |
| Fernandes et al. (2017) | Low risk | Low risk | Low risk | Low risk |
| Takenaka et al. (2023) | Low risk | Low risk | Low risk | Low risk |

Notes: QUADAS-2, Quality Assessment of Diagnostic Accuracy Studies-2.
