# Supplementary material for: Prognostic Value of Enterography Findings in Crohn’s Disease: A Systematic Review and Meta-Analysis
Source: J Imaging. 2025 Nov 5;11(11):392. doi: 10.3390/jimaging11110392 (PMC12653103; doi:10.3390/jimaging11110392)
Supplement: Supplementary file 1 [file jimaging-11-00392-s001.zip › Supplementary File S4.pdf]

Supplementary File S4. . GRADE summary of findings: Inflammation compared with no inflammation in Crohn's disease (prognostic assessment by MRE/CTE)

Certainty assessment							№ of patients		Effect		Certainty	Importance
№ of studies	Study design	Risk of bias	Inconsistency	Indirectness	Imprecision	Other considerations	Inflammation	No inflammation	Relative (95% CI)	Absolute (95% CI)		
**Hospitalization (assessed with: Need for hospitalization related to Crohn's disease)**												
4	non-randomised studies	not serious	serious[a]	not serious	not serious	strong association	122/473 (25.8%)	22/259 (8.5%)	**RR 2.36** (1.23 to 4.52)	**116 more per 1.000** (from 20 more to 299 more)	⨁⨁◯◯ Low[a]	CRITICAL
**Therapeutic escalation (assessed with: Need for therapeutic escalation related to Crohn's disease)**												
4	non-randomised studies	not serious	very serious[b]	not serious	not serious	strong association	237/473 (50.1%)	51/259 (19.7%)	**RR 2.71** (1.18 to 6.25)	**337 more per 1.000** (from 35 more to 1.000 more)	⨁◯◯◯ Very low[b]	CRITICAL
**Surgery (assessed with: Need for abdominal surgery related to Crohn's disease)**												
5	non-randomised studies	not serious	not serious	not serious	not serious	very strong association[c]			**OR 7.42** (2.96 to 18.60)	**7 fewer per 1.000** (from 19 fewer to 3 fewer)	⨁⨁⨁⨁ High[c]	CRITICAL

CI: confidence interval; **OR:** odds ratio; **RR:** risk ratio

Explanations

a. Downgraded one level for inconsistency ($I^2 = 49\%$). Moderate heterogeneity likely arises from heterogeneous definitions of 'inflammation' on MRE/CTE (e.g., MaRIA thresholds vs. qualitative activity, single vs. serial imaging), differences in baseline risk/treatment status across cohorts, and follow-up length. Direction of effect was consistent, but the magnitude varied (study RRs ≈1.08–5.22)

b. Downgraded two levels for inconsistency ($I^2 = 90\%$, $\tau^2 = 0.5852$). Heterogeneity is likely due to differences in study design, definitions of therapeutic escalation, and patient populations. Upgraded one level for a large magnitude of effect (RR 2.71; 95% CI 1.18–6.25).

c. Upgraded two levels for a very large magnitude of effect (OR 7.42; 95% CI 2.96–18.60). No inconsistency ($I^2=0\%$).
