# Supplementary material for: Prognostic Value of Enterography Findings in Crohn’s Disease: A Systematic Review and Meta-Analysis
Source: J Imaging. 2025 Nov 5;11(11):392. doi: 10.3390/jimaging11110392 (PMC12653103; doi:10.3390/jimaging11110392)
Supplement: Supplementary file 1 [file jimaging-11-00392-s001.zip › Supplementary File S5.pdf]

Supplementary File S5. GRADE summary of findings: Stenosis compared with no stenosis in Crohn's disease (prognostic assessment by MRE/CTE).

Certainty assessment							№ of patients		Effect		Certainty	Importance
№ of studies	Study design	Risk of bias	Inconsistency	Indirectness	Imprecision	Other considerations	Stenosis	No stenosis	Relative (95% CI)	Absolute (95% CI)		
**Surgery (assessed with: Need for abdominal surgery related to Crohn's disease)**												
4	non-randomised studies	not serious	serious[a]	not serious	not serious	strong association	82/152 (53.9%)	30/218 (13.8%)	**RR 4.81** (2.17 to 10.67)	**524 more per 1.000** (from 161 more to 1.000 more)	⨁⨁◯◯ Low[a]	CRITICAL

CI: confidence interval; **RR:** risk ratio

Explanations

a. Downgraded one level for inconsistency (I^2 = 78%); direction of effect consistent, but magnitude varied. Sensitivity analysis (leave-one-out) showed robustness and reduced heterogeneity when Schulberg et al. was omitted. Upgraded one level for a large magnitude of effect (RR 4.81; 95% CI 2.17–10.67).
