# Supplementary material for: Prognostic Value of Enterography Findings in Crohn’s Disease: A Systematic Review and Meta-Analysis
Source: J Imaging. 2025 Nov 5;11(11):392. doi: 10.3390/jimaging11110392 (PMC12653103; doi:10.3390/jimaging11110392)
Supplement: Supplementary file 1 [file jimaging-11-00392-s001.zip › Supplementary File S6.pdf]

Supplementary File S6. GRADE summary of findings: Transmural healing compared with no transmural healing in Crohn's disease (prognostic assessment by MRE/CTE).

Certainty assessment							№ of patients		Effect		Certainty	Importance
**№ of studies**	**Study design**	**Risk of bias**	**Inconsistency**	**Indirectness**	**Imprecision**	**Other considerations**	**Transmural healing**	**No transmural healing**	**Relative (95% CI)**	**Absolute (95% CI)**		
**Surgery (follow-up: range 12 months to 30 months; assessed with: Need for abdominal surgery related to Crohn's disease)**												
3	non-randomised studies	not serious	not serious	not serious	not serious	very strong association	1/242 (0.4%)	52/498 (10.4%)	**RR 0.09** (0.02 to 0.38)	**95 fewer per 1.000** (from 102 fewer to 65 fewer)	⨁⨁⨁⨁ High	CRITICAL
**Hospitalization (follow-up: range 12 months to 30 months; assessed with: Need for hospitalization related to Crohn's disease)**												
3	non-randomised studies	not serious	serious[a]	not serious	not serious	very strong association	10/242 (4.1%)	112/498 (22.5%)	**RR 0.17** (0.06 to 0.53)	**187 fewer per 1.000** (from 211 fewer to 106 fewer)	⨁⨁⨁◯ Moderate[a]	CRITICAL

CI: confidence interval; **RR:** risk ratio

Explanations

a. Serious inconsistency ($I^2 = 54\%$). Moderate heterogeneity likely explained by differences in definitions of transmural healing and hospitalization, as well as variation in patient populations and follow-up duration across studies. Downgraded one level for inconsistency ($I^2 = 54\%$). Moderate heterogeneity likely due to differences in definitions of transmural healing and hospitalization, as well as variation in patient populations and follow-up duration across studies. Upgraded two levels for a very large magnitude of effect (RR 0.17; 95% CI, 0.06–0.53).
