# Supplementary material for: Prognostic Value of Enterography Findings in Crohn’s Disease: A Systematic Review and Meta-Analysis
Source: J Imaging. 2025 Nov 5;11(11):392. doi: 10.3390/jimaging11110392 (PMC12653103; doi:10.3390/jimaging11110392)
Supplement: Supplementary file 1 [file jimaging-11-00392-s001.zip › Supplementary File S7.pdf]

Supplementary File S7. PRISMA 2020 Checklist.

Section/Topic	Item	Checklist item	Reported on page/section
TITLE	1	Identify the report as a systematic review.	Title page
ABSTRACT	2	See the PRISMA 2020 for Abstracts checklist.	Abstract (p.1)
INTRODUCTION	3	Rationale: Describe the rationale for the review.	Introduction (p.2)
	4	Objectives: Provide an explicit statement of the objective(s).	Introduction (p.2)
METHODS	5	Eligibility criteria: Specify inclusion/exclusion criteria.	Methods – Eligibility criteria
	6	Information sources: Specify databases, date searched.	Methods – Data sources and search strategy
	7	Search strategy: Present full search strategies.	Supplementary Appendix S1

8	Selection process: Specify how studies were selected.	Methods – Study selection
9	Data collection process: Specify how data were collected.	Methods – Data extraction
10	Data items: List and define all outcomes, variables.	Methods – Data extraction
11	Study risk of bias assessment.	Methods – Methodological quality (QUADAS-2)
12	Effect measures: Specify effect measures used.	Methods – Data synthesis/statistical analysis
13	Synthesis methods: Describe methods to synthesize results.	Methods – Data synthesis/statistical analysis
14	Reporting bias assessment.	Methods – Data synthesis/statistical analysis (funnel plots)
15	Certainty assessment.	Methods – Methodological quality (GRADE)

RESULTS	16	Study selection: Describe number of studies screened and included, with reasons for exclusions.	Results + Figure 1 (PRISMA flow diagram)
	17	Study characteristics.	Supplementary Table S1
	18	Risk of bias in studies.	Supplementary Table S2 (QUADAS-2)
	19	Results of individual studies.	Results – Meta-analysis subsections
	20	Results of syntheses.	Results – Meta-analysis subsections
	21	Reporting biases.	Results – Funnel plots
	22	Certainty of evidence.	Supplementary Table S3 (GRADE)
DISCUSSION	23	Summary of evidence.	Discussion
	24	Limitations of evidence.	Discussion
	25	Conclusions.	Conclusions
OTHER	26	Registration and protocol.	Methods – PROSPERO CRD420250644775

27	Support: Describe sources of support.	Additional Information – Funding
28	Competing interests.	Additional Information – Conflict of Interest
29	Availability of data, code, and other materials.	Additional Information – Data Availability Statement
30	Ethics approval.	Methods
