# Supplementary material for: Prognostic Value of Enterography Findings in Crohn’s Disease: A Systematic Review and Meta-Analysis
Source: J Imaging. 2025 Nov 5;11(11):392. doi: 10.3390/jimaging11110392 (PMC12653103; doi:10.3390/jimaging11110392)
Supplement: Supplementary file 1 [file jimaging-11-00392-s001.zip › Supplementary File S1.pdf]

Supplementary File S1. Search strategy.

The search strategy was developed with the support of controlled descriptors (MeSH and Emtree), synonyms, and free terms related to Crohn's disease, imaging methods (MRE and CTE), and prognostic outcomes. The search was conducted across eight databases: PubMed, PubMed Central (PMC), Scopus, Web of Science, Embase, DECS, Rayyan, and ProQuest, utilizing controlled vocabulary and following term combinations below.

Review question:

Do findings from magnetic resonance enterography or computed tomography enterography predict the prognosis of Crohn's disease?

Main descriptors used:

- Crohn Disease
- Prognosis
- Magnetic Resonance Imaging (MRI)
- Multidetector Computed Tomography (MDCT)
- Enterography
- Magnetic Resonance Enterography (MRE)
- Computed Tomography Enterography (CTE)

Complete PubMed search strategy example:

(((Crohn Disease[MeSH Terms]) OR ("Crohn Disease"[Title/Abstract] OR "Crohn's Disease"[Title/Abstract] OR "Crohns Disease"[Title/Abstract] OR "Crohn's Enteritis"[Title/Abstract] OR "Inflammatory Bowel Disease 1"[Title/Abstract] OR "Regional Enteritis"[Title/Abstract] OR Ileocolitis[Title/Abstract] OR "Terminal Ileitis"[Title/Abstract] OR "Regional Ileitis"[Title/Abstract] OR "Enteritis, Regional"[Title/Abstract] OR "Granulomatous Colitis"[Title/Abstract])) AND ((Prognosis[MeSH Terms]) OR (Prognosis[Title/Abstract] OR Prognoses[Title/Abstract] OR "Prognostic Factors"[Title/Abstract] OR "Prognostic Factor"[Title/Abstract]))) AND (((Magnetic Resonance Imaging[MeSH Terms]) OR ("Magnetic Resonance Imaging"[Title/Abstract] OR MRI[Title/Abstract] OR "NMR Imaging"[Title/Abstract])) OR (Multidetector Computed Tomography[MeSH Terms]) OR ("Multidetector Computed Tomography"[Title/Abstract] OR MDCT[Title/Abstract] OR MSCT[Title/Abstract])) AND (Enterography[Title/Abstract] OR "magnetic resonance enterography"[Title/Abstract] OR "computed tomography enterography"[Title/Abstract] OR "MR-enterography"[Title/Abstract]))

Search dates:

• First search: August 7, 2024

• Expanded search: August 27, 2024

• Review and deduplication in Rayyan: August 28, 2024

Combined search results:

• Initial total records: 1,113

- Records excluded due to duplication: 199
- Total after deduplication: 914
