# Supplementary material for: Prognostic Value of Enterography Findings in Crohn’s Disease: A Systematic Review and Meta-Analysis
Source: J Imaging. 2025 Nov 5;11(11):392. doi: 10.3390/jimaging11110392 (PMC12653103; doi:10.3390/jimaging11110392)
Supplement: Supplementary file 1 [file jimaging-11-00392-s001.zip › Supplementary File S2.pdf]

Supplementary File S2. Characteristics of the studies included in the meta-analysis.

Author	Year	Country/Center	Study Design	N	Population characteristics	Imaging modality	Radiological criteria evaluated	Clinical outcomes evaluated	Follow-up duration	Therapy
Mao et al.	2013	China, First Affiliated Hospital of Sun Yat-Sen Univ.	Prospective cohort	32	CD patients after ileocolic resection were evaluated for postoperative recurrence	CTE was performed postoperatively, within 1 week of colonoscopy	Inflammatory recurrence on CTE (score 0–3, including wall thickening, enhancement, prestenotic dilatation, fistula/abscess)	Surgery	Median 10.5 months (2–30)	Heterogeneous postoperative prophylaxis (5-ASA 31.2%, azathioprine 28%, corticosteroids 15.6%, infliximab 6.2%, no treatment 6.2%)
Gibson et al.	2015	Ireland, St. Vincent's University Hospital	Retrospective cohort	75	Ileal/ileocolonic CD at initiation of anti-TNF, long-standing disease (median 7.9 years)	1.5T MRE, performed within 6 months before anti-TNF initiation	Stricture with prestenotic dilatation (≥80% luminal reduction + wall thickening >3 mm + upstream dilatation)	Surgery	Median 16.7 months (9.0–30.1)	All on anti-TNF (40% infliximab, 60% adalimumab); 53% combined with immunosuppressant

Author	Year	Country/Center	Study Design	N	Population characteristics	Imaging modality	Radiological criteria evaluated	Clinical outcomes evaluated	Follow-up duration	Therapy
Fernandes et al.	2017	Portugal, Hospital de Santa Maria	Retrospective cohort	214	Ileal/ileocolonic CD, 30% perianal, 41% prior surgery	1.5T MRE, performed within 6 months of colonoscopy	Transmural healing: inactive MRE (no wall thickening >3 mm, no enhancement or complications) + inactive endoscopy	Surgery and hospitalization	12 months	The majority on thiopurines (55%); 19% on anti-TNF; a few on methotrexate; many previously exposed to immunosuppressants
Ilias et al.	2018	Hungary, Semmelweis University / Canada, McGill University	Retrospective cohort	75	CD with perianal fistula (56%) and prior surgery (55%)	3T MRE, performed fast-track in referred patients with suspected activity or complication	Active inflammation (thickening, enhancement, edema, ulcers, comb sign, abscess/fistula); significant activity defined by multiple signs or complications	Therapeutic escalation, hospitalization, and surgery	Up to 3 months	Heterogeneous (immunosuppressants, biologics, corticosteroids, antibiotics; not standardized)

Author	Year	Country/Center	Study Design	N	Population characteristics	Imaging modality	Radiological criteria evaluated	Clinical outcomes evaluated	Follow-up duration	Therapy
Mainenti et al.	2020	Italy, Univ. Federico II of Naples	Retrospective cohort	70	Ileal/ileocolonic CD, 46% prior surgery	3T MRE, baseline, patients followed for 1 year	Stricture with prestenotic dilatation >2.5 cm	Surgery	12 months	Heterogeneous regimen (steroids, thiopurines, anti-TNF in part of the cohort; not standardized)
Schulberg et al.	2020	Australia, St Vincent's Hospital / Univ. Melbourne	Retrospective cohort	136	Stricturing CD (84% B2), 67% prior surgery	1.5T MRE, baseline at stricture diagnosis	Stricture with prestenotic dilatation ≥3.0 cm	Surgery	Median 41 months for non-operated; median time to surgery 6 months	68% on immunomodulators, 30% corticosteroids, 28% anti-TNF at diagnosis

Author	Year	Country/Center	Study Design	N	Population characteristics	Imaging modality	Radiological criteria evaluated	Clinical outcomes evaluated	Follow-up duration	Therapy
Hallé et al.	2020	France, CHRU Lille	Retrospective cohort	115	Small bowel CD, 45% prior surgery	1.5T MRE, two serial evaluations within 3–12 months	Active inflammation: non-responders (n=61); wall thickening >7 mm, enhancement, DWI/DCE hyperintensity, comb sign, lymphadenopathy, stricture, abscess, or fistula	Therapeutic escalation and hospitalization	Median 17 months after second MRE (11.6–28.3)	82% exposed to immunosuppressants; 65% on anti-TNF; few on vedolizumab (1%) or ustekinumab (4%)
Oh et al.	2022	Korea, Asan Medical Center	Retrospective cohort	392	CD on anti-TNF ≥1 year	CTE or MRE, performed 1 year after anti-TNF initiation, within ≤3 months of colonoscopy	Active inflammation (wall thickening ≥3 mm, hyperenhancement, mural abnormality, perienteric infiltration, or new/worsened complications); Transmural healing (absence of these findings)	CD-related surgery; therapeutic escalation (anti-TNF intensification or switch); hospitalization for disease activity or treatment complications	Median 18 months (15–21)	All on anti-TNF (infliximab or adalimumab)
Takenaka et al.	2023	Japan, Tokyo Medical and Dental University	Prospective cohort	134	Ileal/ileocolonic CD (71%), 35% prior surgery	3T MRE, performed 1 year after biologic induction, within ≤3 months of colonoscopy	Transmural healing: sMaRIA <2 in most affected segment + endoscopy without activity (SES-CD <4)	Hospitalization and surgery	Median 30 months	All on biologics ≥1 year (49% infliximab, 23% adalimumab, 20% ustekinumab, 8% vedolizumab; 50% combined with immunomodulator)

Author	Year	Country/Center	Study Design	N	Population characteristics	Imaging modality	Radiological criteria evaluated	Clinical outcomes evaluated	Follow-up duration	Therapy
Lu et al.	2024	China, Sun Yat-Sen University Hospital	Retrospective cohort	175	Active CD, mostly ileocolonic (86%), 58% perianal, 23% prior surgery	3T MRE, performed 6–12 months after baseline, within ≤3 months of colonoscopy	Active inflammation: absence of TH. TH is defined as wall thickness ≤3 mm without inflammatory signs (edema, enhancement, ulceration, DWI hyperintensity) and without complications (stricture, abscess, fistula)	Therapeutic escalation, hospitalization, and surgery	Median 17.4 months (11.6–25.5)	Heterogeneous: biologics (anti-TNF 37%, vedolizumab 10%, ustekinumab 12%), immunomodulators 18%, corticosteroids 9%, combination 23%
Fernández-Clotet et al.	2024	Spain, Hospital Clínic de Barcelona	Prospective cohort	89	Ileal/ileocolonic CD, 30% prior surgery	1.5T/3T MRE, performed 46 weeks after initiation of biologic therapy	Stricture with upstream dilatation >3 cm	Surgery	2 years	78% on anti-TNF; others on vedolizumab or ustekinumab; 78% combined with immunosuppressants
